# Combined diagnostic value of NT-proBNP, DLK-1, PSP-D and PCSK-9 in heart failure with preserved ejection fraction: a prospective biomarker study

**DOI:** 10.3389/fcvm.2026.1787516

**Published:** 2026-04-14

**Authors:** Liu Xiaobin, Qin Yu, Feng Jingjie, Liu Chang, Zhang Shuang, Yang Jianing, Wen Jiangping, Niu Yonghong

**Affiliations:** 1 The First Hospital of Tsinghua University, Beijing, China; 2 Department of Medical Genetics and Developmental Biology, School of Basic Medical Sciences, Beijing, China

**Keywords:** DLK-1, heart failure, NT-proBNP, PCSK-9, PSP-D

## Abstract

**Background:**

Heart failure with preserved ejection fraction (HFpEF) remains a formidable clinical challenge due to its intricate pathophysiology and the difficulty in early diagnosis. Traditional biomarkers for HFpEF are often insufficient in clinical practice, driving the need for more novel, sensitive diagnostic markers.

**Methods:**

Serum samples from 58 HFpEF patients and 30 healthy controls were analyzed using Olink proteomics technology. Advanced machine-learning algorithms were employed to comprehensively compare the diagnostic performance of individual biomarkers and their combinations.

**Results:**

The serum levels of DLK-1, PSP-D, and PCSK-9 in HFpEF patients were higher than those in the control group. NT-proBNP, UA and PCSK-9 were identified as risk factors for HFpEF, with regression coefficients of 0.009 for NT-proBNP, 0.006 for UA, and 1.061 for PCSK-9 respectively. The area under the receiver operating characteristic curve (AUC) of the combination of NT-proBNP, DLK-1, PSP-D and PCSK-9 for the diagnosis of HFpEF reached 0.794. This value outperformed the AUC of the combination of PCSK-9 and NT-proBNP, 0.788, the combination of three markers (DLK-1, PSP-D, and PCSK-9, 0.622), as well as the AUCs of each of the four markers alone (NT-proBNP: 0.778, DLK-1: 0.578, PSP-D: 0.523, PCSK-9: 0.628).

**Conclusion:**

The combined detection of NT-proBNP, DLK-1, PSP-D and PCSK-9 can significantly enhance the specificity and sensitivity of the clinical diagnosis of HFpEF patients, holding great potential for improving the diagnostic accuracy of HFpEF in clinical settings.

## Highlights

• For the first time, DLK-1, PSP-D, and PCSK-9 have been used in combination for the diagnosis of patients with preserved ejection fraction.

• The serum levels of DLK-1, PSP-D, and PCSK-9 may serve as supplementary biomarkers for diagnosing HFpEF, and their combined use with NT-proBNP could enhance the diagnostic efficacy for heart failure with preserved ejection fraction.

## 1 Introduction

Heart failure (HF) constitutes a significant global public health challenge. Approximately 64.3 million adults worldwide are affected by HF, while in China, its prevalence among adult ranges from 13% to 18% (1). Heart failure, the terminal stage of cardiac disease, carries a poor prognosis. Its etiology is primarily attributed to three pathophysiological mechanisms: (1) structural and functional abnormalities due to excessive ventricular dilation or impaired contraction; (2) pathological alterations in cardiomyocytes; and (3) elevated natriuretic peptide levels and/or increased cardiac filling pressure (2, 3).

Heart failure is categorized into three types based on the ejection fraction: heart failure with preserved ejection fraction (HFpEF), heart failure with intermediate ejection fraction (heart failure with midrange ejection fraction, HFmrEF) and heart failure with reduced ejection fraction (HFrEF). For HFpEF patients, the left ventricular ejection fraction (LVEF) is 50% or above; for HFmrEF patients, it ranges from 40% to 49%; and for HFrEF patients, it is less than 40%. The diagnosis of HFpEF is often delayed or missed in patients with minimal or no symptoms (4). This diagnostic difficulty, combined with its role as an early-stage condition, makes HFpEF a critical focus for this research.

It is currently understood that the pathogenesis of HFpEF involves impaired left ventricular diastolic function, elevated left ventricular filling pressure, and/or reduced stroke volume, resulting in a decrease in circulating blood volume and tissue perfusion (5). The “diagnostic gold standard” for HF involves exercise testing combined with invasive hemodynamic assessment. In contrast, the routine clinical diagnosis of heart failure is made through a clinical assessment of signs and symptoms, supplemented by measurement of left ventricular ejection fraction in our country (6).

B-type natriuretic peptide (BNP) and N-terminal pro-B-type natriuretic peptide (NT-proBNP) serve as the principal serum biomarkers for the clinical diagnosis and assessment of heart failure. Its interpretation, however, is complicated by several confounding factors, including age, sex, body mass index (BMI), and renal function. In the emergency department, the high sensitivity of natriuretic peptides is achieved by using low “rule-out cut-off values” (e.g., BNP < 100 pg/mL), with the aim of ensuring that no cases are missed. The low specificity of natriuretic peptides may stem from studies conducted in elderly hospitalized patients with multiple comorbidities (such as renal failure, COPD, and atrial fibrillation) using standard cut-off values. This is because these conditions themselves can lead to a significant elevation in natriuretic peptide levels, resulting in a high number of false positives (3). Nevertheless, the diagnostic utility of both natriuretic peptides may be more limited in HFpEF, likely due to its distinct pathophysiology and the characteristically lower range of biomarker elevation in this condition. Therefore, advancing the diagnostic paradigm for HFpEF, by discovering and validating more discriminative biomarkers, is crucial to facilitate early diagnosis, prompt intervention, and better long-term results (4).

This study aimed to identify and evaluate a panel of novel biomarkers for HFpEF. We specifically assessed the diagnostic performance of DLK-1, PSP-D, and PCSK-9.

## 2 Data and methods

### 2.1 Inclusion and exclusion criteria

Fifty-eight HFpEF patients, including 33 men and 25 women aged 55.75–74 years (median age of 66 years), were recruited for this study from the Department of Cardiology of the First Hospital of Tsinghua University from July 2023 to April 2024.

#### 2.1.1 Patients meeting the following criteria were included

(1) The diagnostic criteria in the Chinese Guidelines for the Diagnosis and Treatment of Heart Failure (3); the patient had no corresponding symptoms and/or signs (caused by noncardiac disease, effective treatment was not clearly defined); LVEF was at least 50%, increased natriuretic peptide level and one of the following three, left ventricular hypertrophy, left atrial enlargement or abnormal cardiac diastolic function; and (2) aged 30 years or older.

#### 2.1.2 Patients meeting the following conditions were excluded

(1) Acute endocarditis, myocarditis or pericarditis half a year before admission; (2) acute myocardial infarction (three months before admission) or ST-elevation myocardial infarction four months before admission; (3) acute infectious disease; (4) acute decompensated HF; (5) malignant tumors.

The control group and the experimental group were matched at a 1:2 ratio based on gender and age. Thirty healthy people were used as the control group, including 17 men and 13 women aged 55.75–73.75 years (median age 66 years).

**Figure 1:**
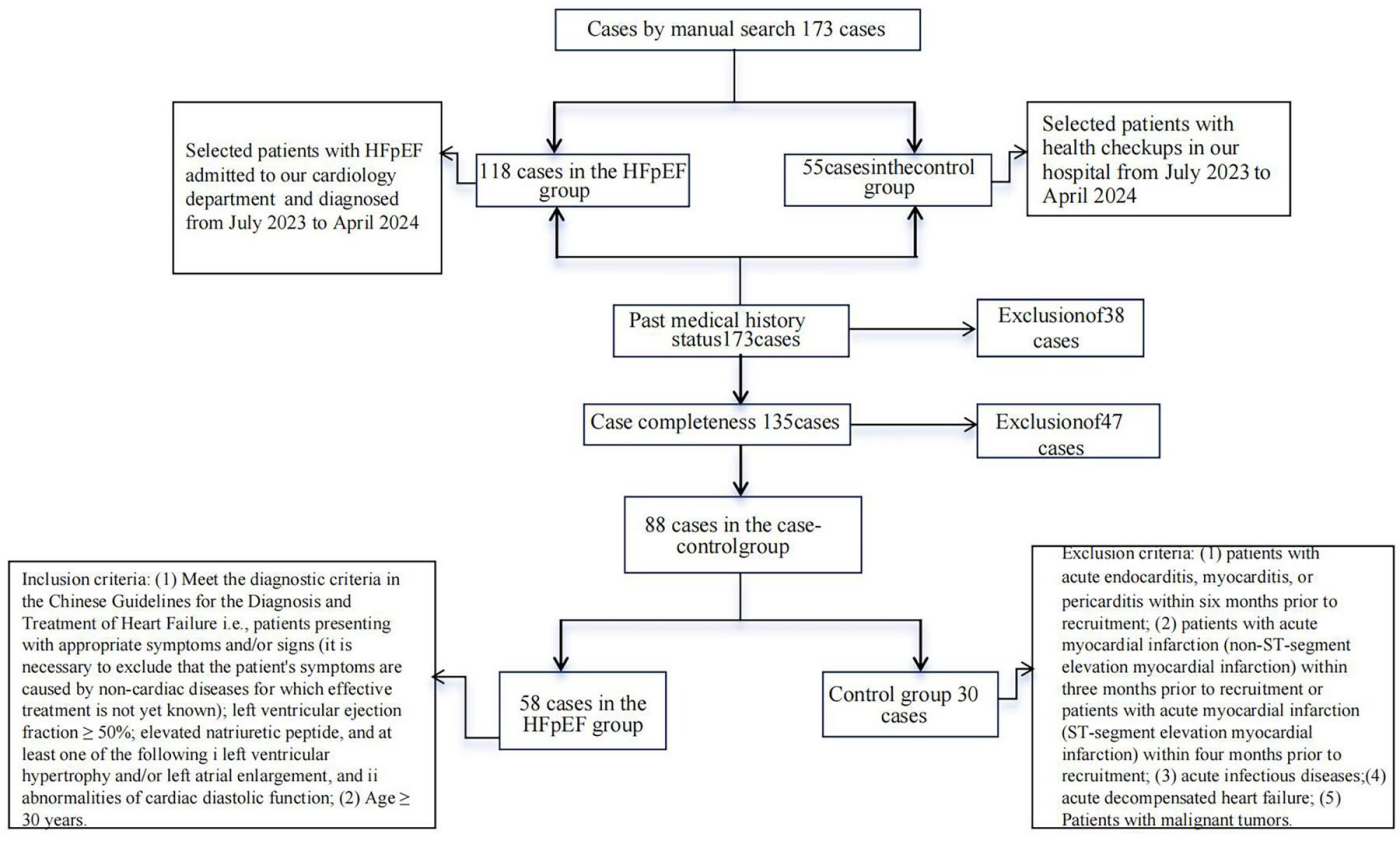
Olink boxplot of all protein biomarkers for HF.

A flow chart of the 88 enrolled participants is shown in Figure 1.

#### 2.1.3 Management of borderline or ambiguous cases

Cases where clinical presentation, biomarker levels, or echocardiographic findings were borderline (e.g., mild elevation of NT-proBNP not reaching the guideline-specified threshold, or equivocal diastolic function parameters), the study implemented a standardized review procedure. Each such case was first independently assessed by two senior cardiologists. If their evaluations disagreed, the case was referred to a core adjudication committee including a third expert for final determination.

### 2.2 Study protocol and measurement method

#### 2.2.1 Blood collection and processing

Five milliliters of venous blood was collected from the enrolled participants before physical examination. The samples were centrifuged at 3,000 r/min for 10 min, and the serum was isolated and stored at −80°C. Biological markers were detected on an M1000 automatic biochemical analyzer (Mindray Medical Company, Beijing). The remaining serum was stored at −80°C for proteomic analysis.

#### 2.2.2 Olink protein screening

Protein marker screening was performed via Olink protein extension technology, in which DNA oligonucleotide-labeled antibody pairs specifically bind to the target antigen in solution, hybridize and are extended by DNA polymerase. The amplified samples were transferred to an integrated microfluidic chip for quantitative PCR (qPCR). The results are presented as the CT value of the qPCR value.

### 2.3 Statistical analysis

#### 2.3.1 Data processing

SPSS 26.0 was used for statistical analysis of the data. Normally distributed data are presented as the means ± standard deviations (x ± s) and were compared with a t test; nonnormally distributed data are presented as the medians (M) and quartiles (P25, P75) and were compared with a nonparametric U test. Logistic regression was used to analyze the influence protein biomarker concentrations on HFpEF. The diagnostic value of DLK-1, PSP-D and PCSK-9 was evaluated via receiver operating characteristic (ROC) curve analysis, and *P* < 0.05 indicated statistical significance.

#### 2.3.2 Protein marker screening

The protein content was accurately measured via the Olink adjacency extension technique, the Ct values of the qPCR data were normalized via NPX Signature software, and Log2 was used to perform logarithmic transformation of the protein expression.

#### 2.3.3 Sample size estimation

According to the purpose and design of the study, the significance level of the hypothesis test was set at 0.05, and the test force was set at 0.8. On the basis of the relevant literature and studies, the following formula was utilized:

$$d\;=\;(M1-M2)/SD_{\mathrm{pooled}}SD_{\mathrm{pooled}}=\sqrt{({\mathrm{SD}}_{1}^{2}+{\mathrm{SD}}_{2}^{2})/2}$$

where SD*_pooled_* is the expected effect size, which was 0.7 in this study. The sample size estimation was carried out via G * Power software. The specific calculation steps were as follows: (1). The *Z* test of two samples was selected. The significance level (ɑ = 0.05) and the test force (1–*β* = 0.80) were input. The expected effect size (q = 0.7) was entered (4). The software calculated the required sample size to be 80 patients: 53 patients in the HFpEF group and 27 patients in the control group. Therefore, at least 80 patients needed to be recruited for this study to ensure that the power of the statistical tests met the expected criteria. To ensure the statistical power of Olink protein screening, 58 patients were included in the HFpEF group, and 30 patients were included in the healthy control group.

## 3 Results

### 3.1 The basic characteristics of the patients in the HFpEF group and control group

There were 33 males in the Heart failure group, accounting for 56.9%, and 17 males in the control group, accounting for 56.7%. There was no statistical difference in gender composition between the two groups. Age-matched controls were utilized, and statistical analysis confirmed no difference in age between the heart failure group and the control group. To account for other potential influences, several variables were compared. The groups showed no significant differences with respect to body temperature (°C), heart rate (beats/min), respiratory rate (breaths/min), systolic pressure (mmHg), and diastolic pressure (mmHg); all measures were comparable across groups (Table 1).

**Table 1:** Baseline data for the heart failure and control groups.

Index	Heart failure group	Control group	*χ* ^2^ */t*	*p*
Men [*n* (%)]	33 (56.9)	17 (56.7)	0.00^b^	0.98
Age (years)	65 ± 12.5	64.7 ± 13.2	0.12^a^	0.90
Body temperature (C°)	36.4 ± 0.5	36.5 ± 0.6	−0.30^a^	0.76
Heart rate (beats/min)	77.7 ± 16.1	83.5 ± 15.9	−1.49^a^	0.14
Breathing rate (breaths/min)	18.9 ± 1.5	18.9 ± 1.9	−0.05^a^	0.96
Systolic pressure (mmHg)	130.38 ± 20.77	130.04 ± 18.9	0.70^a^	0.94
Diastolic pressure (mmHg)	75.43 ± 11.83	76.96 ± 9.96	−0.57^a^	0.57

^a^ Represents the t test and b represents the *χ*^2^ test.

### 3.2 The medical history data of the HFpEF group and control group

The proportion of NYHA class in HFpEF group was 58.6% in grade I, 24.1% in grade II, 5.2% in grade III, 12.1% in grade IV, and the median ejection fraction was 60%. Statistical analysis revealed significant differences between the HFpEF and control groups. A family history of heart disease was more common in the HFpEF group. Furthermore, the prevalences of arrhythmia, coronary artery disease, and stenocardia were all significantly higher in the HFpEF group (all *p* < 0.01) (Table 2).

**Table 2:** Selected medical history data of patients.

Index	Heart failure group	Control group	*χ* ^2^	*p*
NYHA classification [*n* (%)]				
Ⅰ	34 (58.6)	–	–	–
Ⅱ	14 (24.1)	–	–	–
Ⅲ	3 (5.2)	–	–	–
Ⅳ	7 (12.1)	–	–	–
Ejection fraction (%)	60 (56.75,64.25)	–	–	–
Family history of heart disease [*n* (%)]	9 (15.5)	0.000	4.35^a^	<0.05
Glycosuria [*n* (%)]	23 (39.7)	7 (28)	1.03^a^	0.31
Hypertension [*n* (%)]	37 (63.8)	11 (44)	2.81^a^	0.09
Dyslipidemia [*n* (%)]	28 (48.3)	12 (48)	0.00^a^	0.98
Chronic kidney disease [*n* (%)]	3 (5.2)	1 (4)	0.05^a^	0.82
Cerebrovascular disease [*n* (%)]	5 (8.6)	1 (4)	0.56^a^	0.46
Arrhythmia [*n* (%)]	16 (27.6)	0.000	8.54^a^	<0.01
Coronary artery disease [*n* (%)]	39 (67.3)	3 (12)	21.33^a^	<0.01
Stenocardia [*n* (%)]	19 (32.8)	0.000	11.36^a^	<0.01
Myocardial infarction [*n* (%)]	4 (6.9)	0.000	1.81^a^	0.18

^a^ Represents the *χ*^2^ test.

### 3.3 Comparisons of laboratory indicators between the HFpEF and control groups

Laboratory indicators were compared between the two groups. No significant differences were observed in blood cell counts, D-dimer levels, electrolyte profiles and others. Levels of N-terminal pro-B-type natriuretic peptide (NT-proBNP), troponin T, and uric acid (UA) were elevated in the HFpEF group (Table 3).

**Table 3:** Selected laboratory indicators of the patients.

Index	Heart failure group	Control group	*t/z*	*p*
Neutrophil count, Gpt/L	6.86 ± 2.62	5.41 ± 2.33	−0.59^a^	0.56
Lymphocyte count, Gpt/L	1.37 (1.02,1.79)	1.57 (0.94,2.11)	−0.34^b^	0.73
Monocyte count, Gpt/L	0.4 (0.29,0.50)	0.37 (0.32,0.45)	−0.36^b^	0.72
Acidophilic cell count, Gpt/L	0.085 (0.04,0.183)	0.085 (0.04,0.155)	−0.05^b^	0.96
Alkali-prone cell count, Gpt/L	0.025 (0.02,0.04)	0.03 (0.02,0.04)	−1.65^b^	0.10
Erythrocyte count, Tpl/L	4.11 ± 0.68	4.23 ± 0.79	−0.77^a^	0.45
Oxyphorase, g/L	123.07 ± 24.00	129 ± 24.27	−1.09^a^	0.28
Soterocyte, Gpt/L	204 (160.5,250)	219.5 (169,269.5)	−1.06^b^	0.29
D dimer, mg/L	0.16 (0.09,0.29)	0.22 (0.82,0.90)	−1.21^b^	0.22
Creatine kinase MB isoenzyme, ng/mL	1.52 (1.0, 2.74)	1.6 (1.0, 2.1)	−0.33^b^	0.74
Muscle hemoglobin, ng/mL	27.3 (21, 43.7)	16.2 (11.0,40.8)	−1.61^b^	0.11
Troponin T, ng/mL	0.02 (0.01, 0.6)	0.01 (0.005, 0.2)	−2.56^b^	0.01
Potassium, mmol/L	4.0 (3.8, 4.3)	4.1 (3.8, 4.4)	−0.89^b^	0.38
Sodium, mmol/L	139.8 (138,142)	140.3 (138.6,142.4)	−0.53^b^	0.60
Chloride, mmol/L	103.9 (100.4,106.6)	104.4 (101.8,107.1)	−0.90^b^	0.37
Bicarbonate, mmol/L	24.3 (22.8,26.4)	26.4 (25, 29.2)	−2.72^b^	0.07
Total calcium, mmol/L	2.2 ± 0.1	2.2 ± 0.1	2.00^a^	0.05
Magnesium, mmol/L	0.9 ± 0.08	0.9 ± 0.09	−1.60^a^	0.11
Amylaceum, mmol/L	6.0 (5.3,7.7)	6.4 (5.3,9)	−1.00^b^	0.31
Urea, mmol/L	6.3 ± 3.4	6.4 ± 3.0	−0.15^a^	0.88
Creatinine, μmol/L	80 (64,96)	67 (58, 81)	−1.85^b^	0.07
Cystatin C, mg/L	1.0 (0.8,1.3)	1.0 (0.9,1.2)	−0.48^b^	0.63
UA, μmol/L	344.3 ± 110.0	280.2 ± 87.4	2.20^a^	0.03
Total protein, g/L	66.4 ± 5.1	63.5 ± 7.4	2.04^a^	0.05
Albumin, g/L	39.4 ± 4	38.1 ± 7.2	1.10^a^	0.30
Total bilirubin, μmol/L	9.8 (7.2,13.7)	9.7 (7.6,14.6)	−0.06^b^	0.95

^a^ Is the *t* test.

^b^ Is the *U* test.

### 3.4 Biomarkers differentially expressed between HFpEF patients and healthy controls

#### 3.4.1 HFpEF patients showed increased expression of DLK-1, PSP-D, and PCSK-9 relative to the control group, as measured by olink

**Figure 2:**
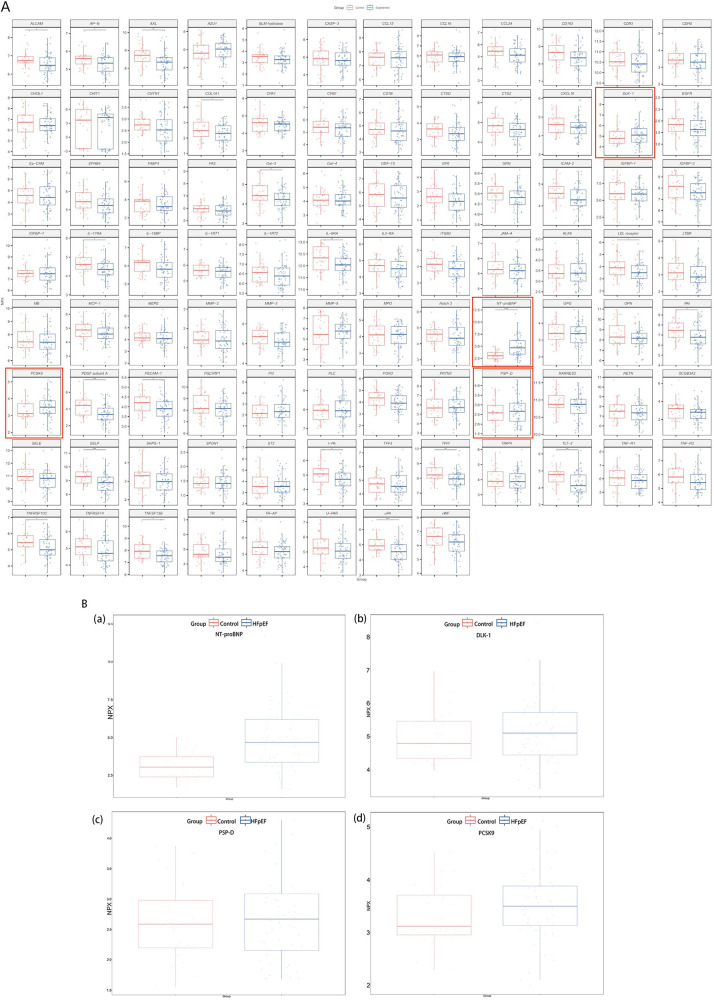
**(A)** Olink screening for cardiac-related protein biomarkers; a detailed list of the 92 proteins is provided in Supplementary Table S2. **(B)** Four protein biomarkers were upregulated in the HFpEF group compared with the control group: **(a)** NT-proBNP; **(b)** DLK-1; **(c)** PSP-D; **(d)** PCSK-9.

The results of the analysis using OLINK technology on patient samples enrolled in the study, which identified heart failure-associated proteins, are presented in Figures 2A,B presents the four proteins elevated in the heart failure group compared to the control group. Figure 2A showed all HFpEF-related proteins screened by OLink; markers higher than those in the control group are circled in red boxes. In Figure 2B, panel a represented NT-proBNP, panel b represented DLK-1, panel c represented PSP-D, and panel d represented PCSK-9. The levels of DLK-1, PSP-D and PCSK-9 were all higher in the HFpEF group than in the control group (Figure 2).

#### 3.4.2 Differential protein biomarkers between HFpEF and control groups

Cardiac markers, including NT-proBNP, creatine kinase MB isoenzyme (CK-MB), myoglobin (MYO), and cardiac troponin T (cTnT), were rapidly measured in HFpEF patients. The HFpEF group had significantly elevated NT-proBNP and cTnT levels compared with controls (*P* < 0.05). Levels of DLK-1, PSP-D, and PCSK-9 tended to be higher in the HFpEF group than in controls, as suggested by the box plots; however, none of these elevations were statistically significant. The difference in PCSK-9 was of marginal significance (*P* = 0.078), as detailed in Table 4.

**Table 4:** Comparison of differentially expressed cardiac-related indicators between the two groups.

Biomarker	Heart failure group	Control group	*t/z*	*p*
NT-proBNP, ng/L	300 (77.8,661.9)	61.09 (31,118.8)	−3.99^b^	0.00**
CK-MB, ng/mL	1.52 (1.0,2.74)	1.6 (1.0,2.1)	−0.33^b^	0.74
MYO, ng/mL	27.3 (21,43.7)	16.2 (11.0,40.8)	−1.61^b^	0.11
cTnT, ng/mL	0.02 (0.01,0.6)	0.01 (0.005,0.2)	−2.56^b^	0.00**
DLK-1 (NPX value)	5.177 ± 0.987	4.929 ± 0.735	−1.21^a^	0.23
PSP-D (NPX value)	2.682 ± 0.667	2.644 ± 0.631	−0.26^a^	0.80
PCSK-9 (NPX value)	3.549 ± 0.713	3.283 ± 0.538	−1.79^a^	0.078
NT-proBNP (NPX value)	5.027 ± 2.430	3.127 ± 0.955	−4.11^a^	0.000***

NT-proBNP, N-terminal B-type natriuretic peptide; CK-MB, creatine kinase MB isoenzyme; MYO, myoglobin; cTnT, troponin T.

^a^ Is the *t* test.

^b^ is the *U* test.

* *p* < 0.05, **p < 0.01, ***p < 0.001.

### 3.5 Factors associated with HFpEF identified by multivariable regression

**Figure 3:**
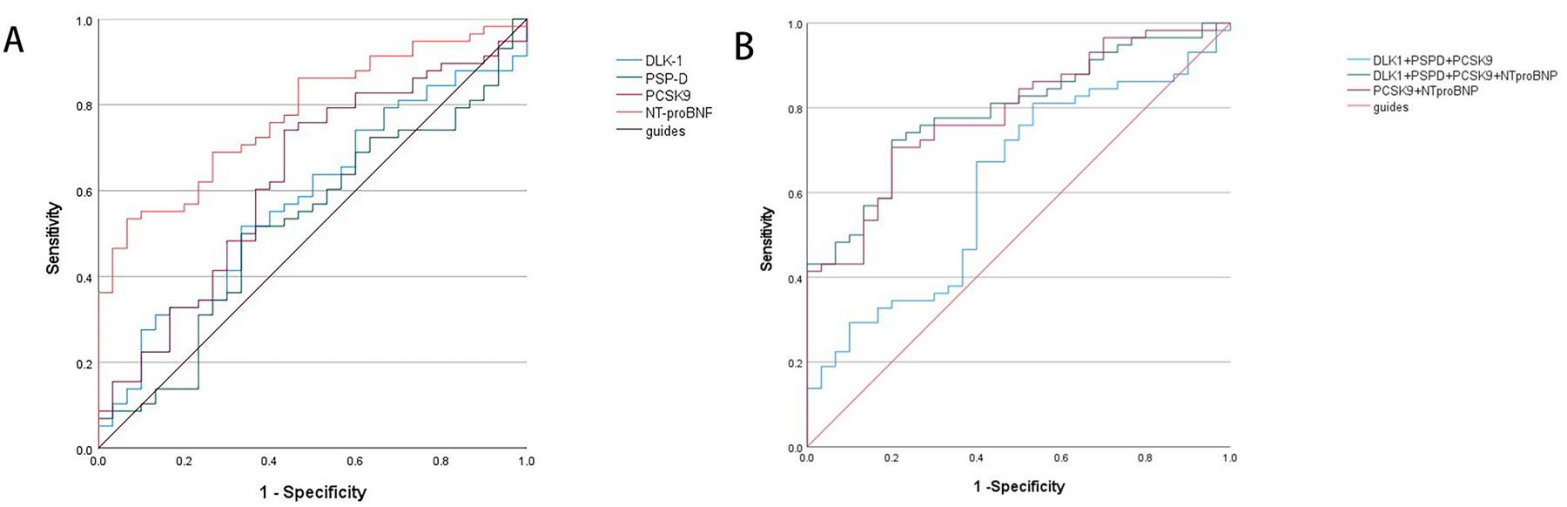
**(A)** ROC curve for single protein biomarkers. **(B)** ROC curve for the combination of protein biomarkers.

A univariate binary logistic regression analysis was conducted, using the presence of HFpEF as the dependent variable and each biomarker as an independent variable (shown in Supplementary Table S1). The meaningful indicators of univariate regression analysis were included in the binary logistic regression analysis and the backward step method. The results revealed that NT-proBNP, was the risk factors for HFpEF, and the regression coefficients were 0.009 for NT-proBNP (Table 5). Each increase of one unit for NT-proBNP was associated with a 0.7% increased risk of HFpEF. 3.6 ROC curve analysis was conducted to evaluate the diagnostic performance of biomarkers for HFpEF (Table 6; Figure 3).

**Table 5:** Logistic regression of the factors influencing HFpEF.

Variable	Regression coefficient	Standard error	OR	95% CI	*P*
PCSK-9	1.061	0.642	2.890	0.822–10.163	0.098
UA	0.006	0.004	1.006	0.99–1.014	0.08
NT-proBNP	0.009	0.003	1.009	1.003–1.016	0.006
Constant	−6.139	2.62	0.002		

OR, odds ratio; CI, confidence interval; NT-proBNP, N-terminal B-type natriuretic peptide; UA, uric acid.

**Table 6:** ROC curve analysis results for variable combinations for the diagnosis of HFpEF.

Variable	AUC	Sensitivity	Specificity	Youden index
DLK-1	0.578	0.517	0.667	0.184
PSP-D	0.523	0.500	0.667	0.167
PCSK-9	0.628	0.741	0.567	0.308
NT-proBNP	0.778	0.534	0.933	0.468
DLK-1 + PSP-D + PCSK-9	0.622	0.810	0.467	0.277
DLK-1 + PSP-D + PCSK-9 + NT-proBNP	0.794	0.724	0.800	0.524
PCSK-9 + NT-proBNP	0.788	0.707	0.800	0.507

HFpEF, heart failure with preserved ejection fraction; AUC, area under the receiver operating characteristic curve; ROC, receiver operating characteristic; NT-proBNP, N-terminal B-type natriuretic peptide.

The 2018 diagnostic and treatment criteria for HF were used as the gold standard for the diagnosis of HFpEF. The natural log values of the expression levels of DLK-1, PSP-D, and PCSK-9 were used as test variables. Patients with HFpEF were used as the case group (positive event), and healthy subjects, according to the guidelines, were used as the control group (negative events). Status variables were used to establish the ROC curve shown in Figure 3A. The results revealed that DLK-1 had an AUC of 0.578, a Youden index of 0.184, a specificity of 0.667, and a sensitivity of 0.517; PSP-D had an AUC for distinguishing HFpEF patients from healthy individuals of 0.523, a Youden index of 0.167, a specificity of 0.667, and a sensitivity of 0.500; and PCSK-9 had an AUC for distinguishing HFpEF patients from healthy individuals of 0.628, a Youden index of 0.308, a specificity of 0.567 and a sensitivity of 0.741. The AUC of NT-proBNP for the diagnosis of HFpEF was 0.778, a Youden index of 0.468, a specificity of 0.933 and a sensitivity of 0.534.

To improve the detection capability, the natural log values of DLK-1, PSP-D, PCSK-9 and NT-proBNP were combined as test variables. With the selected HFpEF patients as the case group (positive events) and healthy examination subjects according to the guidelines as the control group (negative events), the status variables in Table 6 were used to generate the ROC curves in Figure 3B. The AUC of DLK-1, PSP-D, PCSK-9 and NT-proBNP combinition was 0.794, the sensitivity was 0.724, and specificity were 0.800. The AUC of DLK-1, PSP-D and PCSK-9 combination was 0.622, the sensitivity was 0.810, and the specificity was 0.467. The AUC of PCSK-9 and NT-proBNP combination was 0.788, the sensitivity was 0.707, and the specificity was 0.800. Among all models tested, the combined detection of the four proteins achieved the highest AUC, with full statistical results presented in Table 6.

## 4 Discussion

The serum levels of DLK-1, PSP-D and PCSK-9 in HFpEF patients were higher than those in the control group. Also PCSK-9, NT-proBNP, UA were all risk factors for HFpEF. The test demonstrates excellent sensitivity and specificity of the combination of NT-proBNP, DLK-1, PSP-D and PCSK-9 for the diagnosis of HFpEF. This approach compensates for the limitation of the relatively low sensitivity of NT-proBNP when it is detected alone. As such, it can serve as a detection panel for the early diagnosis of HFpEF. It is also worth noting that the specificity of NT-proBNP alone is higher than that of combined detection, making it suitable for exclusion diagnosis in emergency patients to reduce the false-positive rate.

### 4.1 Description of diagnostic criteria

This study was initiated in 2022, and the enrollment and diagnostic classification of patients followed the clinical practices and national guidelines prevalent in China at that time. At that stage, the diagnosis of HFpEF in China relied on a comprehensive assessment integrating clinical symptoms, biomarker profiles, and echocardiographic evidence, rather than mandating the calculation of the HFA-PEFF or H^2^FPEF scores.

We explicitly acknowledge the differences between the diagnostic criteria used in the current research and currently established international scoring systems. To improve the comparability of our findings with international research, we conduct a *post hoc* characterization of the enrolled cohort using detailed individual data reported in the study—such as BMI, atrial fibrillation history, blood pressure, and echocardiographic parameters—to estimate the distribution of patients’ probability of meeting the H^2^FPEF score threshold, and this serves as a reference for cross-study comparison.

### 4.2 Analysis of the clinical data of HFpEF patients and healthy controls

The baseline data showed no significant differences between the HFpEF and control groups in this study. And more patients in the HFpEF group had a family history of heart disease, which was consistent with previous studies (7). Furthermore, studies indicate that atrial fibrillation (AF), the most common arrhythmia, is a significant independent predictor of poorer health-related quality of life (8). Long-term myocardial ischemia and myocardial infarction lead to myocardial fibrosis and myocardial hypofunction, which cause cardiac insufficiency (8–10). Angina pectoris is a manifestation of coronary artery disease, and myocardial ischemia and injury from angina pectoris can exacerbate HF. The HFpEF group included more patients with a history of angina pectoris (11, 12) than the control group. Many previous studies have shown that the occurrence of HF is often associated with hypertension, diabetes, myocardial infarction, stenocardia, coronary artery disease and stenocardia; This corroborates some previous work (11, 13).

NT-proBNP, CK-MB, and cTnT are cardiac markers. NT-proBNP is generally recommended as the preferred marker for HF diagnosis, but its levels and progression are strongly affected by sex and age or other underlying diseases. Therefore, the clinical diagnosis of HFpEF requires a comprehensive evaluation that combines other clinical indicators and patient conditions (14). cTnT has an important role in the diagnosis of unstable angina and acute myocardial infarction and is also important in the diagnosis of heart failure (15). In the present study, patients with HFpEF exhibited significantly higher levels of NT-proBNP and cTnT compared to controls., which is consistent with previous findings.

An increasing number of studies have shown that the heart and kidney are intimately interconnected in the regulation of systemic fluid volume and blood pressure homeostasis. Thus, impaired renal function is an independent risk factor for increased mortality in chronic HF patients (3, 16). UA is the final product of purine metabolism in the body. Elevated UA in the blood can lead to endothelial hyperplasia, oxidative stress, and increased risks of hypertension and angina, which can lead to heart failure (17). While serum UA levels differed significantly between the HFpEF and control groups, the levels of Crea, Urea, and cystatin C did not. A robust interpretation of cardiac function based on these parameters, however, requires careful consideration of the potential influence of renal impairment.

### 4.3 Associations between DLK-1, PSP-D, and PCSK-9 and HF

DLK-1 is a transmembrane protein with multiple biological functions and belongs to the epidermal growth factor (EGF)-like repeat sequence family. During the embryonic period, DLK-1 is widely expressed in neuroendocrine regions such as the pituitary gland and hypothalamus, participating in the regulation of embryonic development (18, 19). In adulthood, it is mainly expressed in neuroendocrine tissues such as the pituitary gland, adrenal gland, pancreas, and dopaminergic neurons in the substantia nigra; it is also expressed in liver stem cells and adipocyte precursor cells, maintaining tissue homeostasis (20, 21). As a transmembrane protein, its EGF-like domain may be involved in intercellular signal transduction. Studies have shown that DLK-1 may affect cardiac remodeling in a myocardial fibrosis model by regulating fibroblast activation and extracellular matrix deposition, indirectly inducing heart failure (22); in lipid metabolism, DLK-1 inhibits adipocyte differentiation, and obesity and metabolic syndrome are important risk factors for heart failure (especially heart failure with preserved ejection fraction, HFpEF) (23, 24). In heart failure research, DLK-1 belongs to the Notch ligand family, and the Wnt signaling pathway (such as DKK-1) has been confirmed to be related to coronary heart disease and heart failure (25); DLK-1 may also indirectly participate in the progression of heart failure by affecting endothelial cell function, similar to the role of HK1 mitochondrial dissociation in HFpEF (26, 27). However, there is currently no direct evidence that DLK-1 is involved in the pathological process of heart failure, its role in fibrosis and metabolic regulation suggests that further exploration is needed to determine whether it affects cardiac function by regulating cardiac fibroblasts or fat metabolism, and whether it is related to microvascular dysfunction in heart failure subtypes (such as HFpEF). In this study, the content of DLK-1 in the HFpEF group was higher than that in the healthy control group, which is consistent with the conclusions of previous studies, but the specific regulatory mechanism still requires further in-depth research.

PSP-D is a collagen calcium-dependent agglutinin secreted by type II alveolar epithelial cells (28). It belongs to the large molecular hydrophilic proteins in pulmonary surfactant (PS) and plays a crucial role in the innate immune defense, inflammation regulation, and maintenance of alveolar homeostasis in the lungs (29, 28). In the research on the relationship between PSP-D and heart failure, there are mainly the following three pulmonary-heart interaction mechanisms. (1)Left ventricular diastolic dysfunction: diseases such as hypertension or diabetes can simultaneously cause abnormal left ventricular diastolic function and elevated PSP-D levels, suggesting that PSP-D may serve as an indirect indicator of systemic inflammation or lung injury (30). (2) As an auxiliary diagnostic marker for heart failure. Heart failure combined with pulmonary diseases: Heart failure patients often have pulmonary congestion or interstitial pulmonary edema, which may lead to increased PSP-D release, but its specificity is low. It needs to be combined with other indicators (such as BNP, NT-proBNP) for comprehensive assessment (31). (3)Metabolic and energy disorders: heart failure patients often have abnormal energy metabolism (such as fatty acid oxidation disorders), and PSP-D may indirectly affect myocardial energy supply by regulating inflammatory responses (32).

The study revealed increased PSP-D levels in the HFpEF group vs. controls. Notably, when used in conjunction with NT-proBNP assessment, PSP-D boosted the diagnostic sensitivity of NT-proBNP for HFpEF.

PCSK-9 (Proprotein Convertase Subtilisin/Kexin Type 9) is a secreted serine protease synthesized by the liver and plays a crucial role in cholesterol metabolism regulation (33). Its core function is to promote the lysosomal degradation of low-density lipoprotein receptor (LDLR) by binding to it, thereby reducing the liver's clearance of low-density lipoprotein cholesterol (LDL-C) and leading to an increase in LDL-C levels in the blood (34). In studies related to the association of PCSK-9 with heart failure, the regulation of cardiac metabolism and inflammation and endothelial function are relatively clear. Among these mechanisms, the absence of PCSK-9 may affect cardiac lipid metabolism through non-LDLR-dependent pathways, resulting in the occurrence and development of heart failure with preserved ejection fraction (HFpEF) (35); on the other hand, PCSK-9 inhibitors can delay the progression of heart failure by inhibiting pro-inflammatory factors (such as TNF-α, IL-6) and improving vascular endothelial function (36). In this study, PCSK-9 was slightly elevated in the HFpEF group, which may indicate that PCSK-9 is the dominant factor involved in cardiac lipid metabolism.

Levels of DLK-1, PSP-D, and PCSK-9 were observed to be elevated in patients with HFpEF relative to controls. However, the observed elevation did not reach statistical significance, potentially because patients were in the early stages of heart failure at the time of testing. Continuous monitoring of these markers to delineate their dynamic changes with disease progression may help establish their definitive clinical utility in heart failure diagnosis.

### 4.4 Analysis of the risk factors for HFpEF

The risk factors for HFpEF in this study was NT-proBNP. As a gold-standard biomarker, NT-proBNP rises early in the course of heart failure and correlates with disease risk, with each incremental unit associated with a graded increase in HF probability (37, 38). Our findings confirm NT-proBNP as an independent risk factor for HFpEF and demonstrate its robust contribution to the logistic regression model, thereby strengthening the established evidence base.

It is notable that the significant associations of UA and PCSK-9 with HFpEF observed in univariate analysis were not retained in the multivariate regression model. This is because each variable in the univariate analysis is independently assessed for its association with HFpEF, meaning their individual contributions are not diluted by other factors. However, when these correlated variables are included together in a multivariate model, the model struggles to determine which one truly drives the outcome. As they compete for explanatory power, the statistical significance of each may diminish, often resulting in non-significant *p*-values (*p* > 0.05) for previously significant variables. Thus, the loss of significance for UA and PCSK-9 in the multivariate model is likely accounted for their strong correlation with NT-proBNP, whose inclusion in the model may largely account for the effects initially attributed to UA and PCSK-9. Alternatively, UA and PCSK-9 might act as intermediate factors along the causal pathway to HFpEF rather than serving as direct drivers. These findings underscore the value of multivariate analysis in accounting for confounding effects and distinguishing truly independent predictors.

### 4.5 ROC curve analysis of the combined tests for the diagnosis of HFpEF

We analyzed the diagnostic performance of DLK-1, PSP-D, and PCSK-9 alone and in combination with NT-proBNP. We found that the AUC of DLK-1, PSP-D, PCSK-9 and NT-proBNP combination was better than those of DLK-1, PSP-D, PCSK-9 alone or the combination of the three before. The combination of DLK-1, PSP-D, and PCSK-9 represents a novel multi-biomarker strategy for HFpEF. Although rarely explored, it holds significant potential for improving early diagnostic sensitivity; The early diagnosis of HFpEF is pivotal for facilitating timely interventions that improve quality of life. Improved diagnostic accuracy enables clinicians to institute targeted strategies earlier—such as optimizing guideline-directed medical therapy and lifestyle interventions—which can potentially curb disease progression and improve long-term outcomes.

### 4.6 Potential biomarkers: clinical implications and limitations

Although DLK-1, PSP-D, and PCSK-9 showed potential to enhance the diagnostic sensitivity of NT-proBNP for HFpEF in the initial analysis, significant clinical limitations remain. The lack of statistical significance relative to NT-proBNP may stem from limited sample size or shared pathophysiological pathways such as myocardial stretch. Additionally, the moderate AUC values currently preclude their use as standalone diagnostic tools. Consequently, the clinical value of these biomarkers likely resides not in replacing NT-proBNP, but in offering new insights into HFpEF pathogenesis and potentially contributing to future multi-marker panels alongside NT-proBNP for improved patient stratification or subtyping.

## 5 Conclusions

The combination of DLK-1, PSP-D, PCSK-9, and NT-proBNP demonstrates superior diagnostic efficacy for HFpEF compared to any alternative detection strategy. The biomarker panel consisting of DLK-1, PSP-D, and PCSK-9 significantly enhances sensitivity in the early detection of HFpEF. Furthermore, the multi-biomarker panel (DLK-1, PSP-D, PCSK-9, and NT-proBNP) addresses the inherent limitation of NT-proBNP alone in detecting early-stage heart failure. This approach establishes an evidence-based framework to guide clinicians in timely diagnosis and intervention, paving the way for improved patient outcomes. Nevertheless, while these molecules represent promising research biomarkers, their routine clinical application still requires further validation through larger prospective studies.

## 6 Limitation

The generalizability of this study's findings is constrained by its limited sample size and the inclusion of a population with early-stage or compensated heart failure. In this clinical context, the incremental diagnostic value of the target biomarkers over established indicators was limited. Future studies with larger, more heterogeneous cohorts are needed for robust validation. Thus, the clinical translation of these results warrants a cautious approach.

## Data Availability

The datasets presented in this study can be found in online repositories. The names of the repository/repositories and accession number(s) can be found in the article/Supplementary Material.
