# Supplementary material for: Combined diagnostic value of NT-proBNP, DLK-1, PSP-D and PCSK-9 in heart failure with preserved ejection fraction: a prospective biomarker study
Source: Front Cardiovasc Med. 2026 Apr 14;13:1787516. doi: 10.3389/fcvm.2026.1787516 (PMC13121242; doi:10.3389/fcvm.2026.1787516)
Supplement: Supplementary file 1 [file Table1.doc]

Table 1

| Variable | Regression coefficient | Standard error | OR | 95% CI | *P* |
| --- | --- | --- | --- | --- | --- |
| gender | -0.09 | 0.454 | 0.991 | 0.407-2.412 | 0.984 |
| age | 0.002 | 0.018 | 1.002 | 0.968-1.038 | 0.902 |
| T | -0.14 | 0.456 | 0.869 | 0.356-2.124 | 0.758 |
| P | -0.021 | 0.015 | 0.979 | 0.951-1.007 | 0.146 |
| R | -0.007 | 0.15 | 0.993 | 0.74-1.333 | 0.963 |
| Bph | 0.001 | 0.012 | 1.001 | 0.978-1.025 | 0.943 |
| Bpl | -0.012 | 0.022 | 0.988 | 0.947-1.030 | 0.569 |
| NEUT | -0.053 | 0.088 | 0.949 | 0.798-1.128 | 0.552 |
| LYMPH | -0.203 | 2.77 | 0.816 | 0.474-1.405 | 0.464 |
| MONO | 0.881 | 1.358 | 2.414 | 0.169-34.535 | 0.516 |
| EO | -1.482 | 1.631 | 0.227 | 0.009-5.556 | 0.364 |
| BASO | -3.213 | 6.509 | 0.04 | 0-13959.252 | 0.622 |
| RBC | -0.247 | 0.32 | 0.781 | 0.417-1.463 | 0.441 |
| HGB | -0.011 | 0.01 | 0.989 | 0.97-1.009 | 0.275 |
| PLT | -0.006 | 0.003 | 0.994 | 0.988-1.001 | 0.76 |
| DD | -0.545 | 0.287 | 0.58 | 0.33-1.018 | 0.058 |
| CKMB | 0.059 | 0.112 | 1.06 | 0.852-1.32 | 0.601 |
| MYO | -0.005 | 0.005 | 0.995 | 0.986-1.004 | 0.315 |
| TNT | 21.032 | 12.928 | 1361820515 | 0.013-1.375E20 | 0.104 |
| K | -0.277 | 0.592 | 0.758 | 0.238-2.418 | 0.64 |
| Na | -0.063 | 0.061 | 0.939 | 0.832-1.059 | 0.303 |
| Cl | -0.075 | 0.057 | 0.927 | 0.83-1.037 | 0.184 |
| HCO | -0.187 | 0.081 | 0.829 | 0.707-0.973 | 0.021 |
| Ca | 3.094 | 1.889 | 22.054 | 0.544-894.349 | 0.102 |
| Mg | -4.607 | 2.882 | 0.01 | 0-2.831 | 0.11 |
| GLU | -0.057 | 0.07 | 0.945 | 0.823-1.084 | 0.419 |
| Urea | -0.019 | 0.017 | 0.981 | 0.853-1.128 | 0.981 |
| Crea | 0.016 | 0.01 | 1.016 | 0.997-1.035 | 0.105 |
| Cysc | 0.208 | 0.68 | 0.759 | 0.325-4.667 | 0.759 |
| UA | 0.007 | 0.003 | 1.007 | 1-1.013 | 0.038 |
| TP | 0.038 | 0.035 | 1.039 | 0.97-1.112 | 0.28 |
| ALB | 0.012 | 0.041 | 1.012 | 0.934-1.097 | 0.767 |
| CHO | -0.104 | 0.142 | 0.901 | 0.682-1.191 | 0.465 |
| TG | 0.044 | 0.31 | 1.045 | 0.569-1.917 | 0.887 |
| HDL | -0.631 | 0.819 | 0.532 | 0.107-2.651 | 0.441 |
| LDL | -0.357 | 0.249 | 0.7 | 0.43-1.139 | 0.151 |
| ApoA | -0.97 | 1.061 | 0.379 | 0.047-3.032 | 0.361 |
| ApoB | -1.137 | 0.914 | 0.321 | 0.053-1.924 | 0.213 |
| TBIL | 0.013 | 0.034 | 1.013 | 0.947-1.082 | 0.713 |
| DBIL | 0.06 | 0.077 | 1.062 | 0.913-1.234 | 0.437 |
| ALT | 0.007 | 0.011 | 0.5 | 0.986-1.029 | 0.5 |
| AST | 0.004 | 0.006 | 1.004 | 0.993-1.015 | 0.441 |
| ALP | -0.021 | 0.009 | 0.98 | 0.963-0.996 | 0.018 |
| GGT | -0.001 | 0.001 | 0.999 | 0.996-1.002 | 0.383 |
| CHE | 0.055 | 0.129 | 1.057 | 0.82-1.362 | 0.67 |
| LD | 0.003 | 0.002 | 1.003 | 0.998-1.007 | 0.304 |
| CRP | 0.004 | 0.008 | 1.004 | 0.987-1.021 | 0.655 |
| KET | -1.119 | 0.721 | 0.327 | 0.079-1.343 | 0.121 |
| UGLU | -0.83 | 0.497 | 0.436 | 0.165-1.154 | 0.095 |
| ULE | -1.379 | 0.532 | 0.252 | 0.089-0.715 | 0.01 |
| UBLD | -0.585 | 0.494 | 0.557 | 0.211-1.468 | 0.237 |
| URBC | -0.002 | 0.002 | 0.998 | 0.995-1.001 | 0.195 |
| UWBC | -0.003 | 0.002 | 0.997 | 0.994-1 | 0.098 |
| NTproBNPc | 0.007 | 0.002 | 1.007 | 1.002-1.012 | 0.003 |
| uPA | 0.286 | 0.276 | 1.331 | 0.775-2.284 | 0.3 |
| npxDLK-1 | 0.322 | 0.268 | 1.379 | 0.816-2.33 | 0.229 |
| npxPSP-D | 0.090 | 0.349 | 1.094 | 0.552-2.168 | 0.796 |
| NpxPCSK9 | 0.652 | 0.374 | 1.92 | 0.922-3.997 | 0.081 |
