# Supplementary material for: Combined diagnostic value of NT-proBNP, DLK-1, PSP-D and PCSK-9 in heart failure with preserved ejection fraction: a prospective biomarker study
Source: Front Cardiovasc Med. 2026 Apr 14;13:1787516. doi: 10.3389/fcvm.2026.1787516 (PMC13121242; doi:10.3389/fcvm.2026.1787516)
Supplement: Supplementary file 2 [file Table2.docx]

Table 2

| Aminopeptidase N (AP-N) | P15144 | Insulin-like growth factor-binding protein 1 (IGFBP-1) | P08833 |
| --- | --- | --- | --- |
| Azurocidin (AZU1) | P20160 | Insulin-like growth factor-binding protein 2 (IGFBP-2) | P18065 |
| Bleomycin hydrolase (BLM hydrolase) | Q13867 | Insulin-like growth factor-binding protein 7 (IGFBP-7) | Q16270 |
| Cadherin-5 (CDH5) | P33151 | Integrin beta-2 (ITGB2) | P05107 |
| CarboxypeptidaseA1 (CPA1) | P15085 | Intercellular adhesion molecule 2 (ICAM-2) | P13598 |
| Carboxypeptidase B (CPB1) | P15086 | Interleukin-1 receptor type 1 (IL-1RT1) | P14778 |
| Caspase-3 (CASP-3) | P42574 | Interleukin-1 receptor type 2 (IL-1RT2) | P27930 |
| Cathepsin D (CTSD) | P07339 | Interleukin-2 receptor subunit alpha (IL2-RA) | P01589 |
| Cathepsin Z (CTSZ) | Q9UBR2 | Interleukin-6 receptor subunit alpha (IL-6RA) | P08887 |
| C-C motif chemokine 15 (CCL15) | Q16663 | Interleukin-17 receptor A (IL-17RA) | Q96F46 |
| C-C motif chemokine 16 (CCL16) | O15467 | Interleukin-18-binding protein (IL-18BP) | O95998 |
| C-C motif chemokine 24 (CCL24) | O00175 | Junctional adhesion molecule A (JAM-A) | Q9Y624 |
| CD166 antigen (ALCAM) | Q13740 | Kallikrein-6 (KLK6) | Q92876 |
| Chitinase-3-like protein 1 (CHI3L1) | P36222 | Low-density lipoprotein receptor (LDL receptor) | P01130 |
| Chitotriosidase-1 (CHIT1) | Q13231 | Lymphotoxin-beta receptor (LTBR) | P36941 |
| Collagen alpha-1(I) chain (COL1A1) | P02452 | Matrix extracellular phosphoglycoprotein (MEPE) | Q9NQ76 |
| Complement component C1q receptor (CD93) | Q9NPY3 | Matrix metalloproteinase-2 (MMP-2) | P08253 |
| Contactin-1 (CNTN1) | Q12860 | Matrix metalloproteinase-3 (MMP-3) | P08254 |
| C-X-C motif chemokine 16 (CXCL16) | Q9H2A7 | Matrix metalloproteinase-9 (MMP-9) | P14780 |
| Cystatin-B (CSTB) | P04080 | Metalloproteinase inhibitor 4 (TIMP4) | Q99727 |
| Elafin (PI3) | P19957 | Monocytechemotactic protein 1 (MCP-1) | P13500 |
| Ephrin type-B receptor 4 (EPHB4) | P54760 | Myeloblastin (PRTN3) | P24158 |
| Epidermal growth factor receptor (EGFR) | P00533 | Myeloperoxidase (MPO) | P05164 |
| Epithelial cell adhesion molecule (Ep-CAM) | P16422 | Myoglobin (MB) | P02144 |
| E-selectin (SELE) | P16581 | Neurogenic locus notch homolog protein 3 (Notch 3) | Q9UM47 |
| Fatty acid-binding protein, adipocyte (FABP4) | P15090 | N-terminal prohormone brain natriuretic peptide (NT-proBNP) | NA |
| Galectin-3 (Gal-3) | P17931 | Osteopontin (OPN) | P10451 |
| Galectin-4 (Gal-4) | P56470 | Osteoprotegerin (OPG) | O00300 |
| Granulins (GRN) | P28799 | Paraoxonase (PON3) | Q15166 |
| Growth/differentiation factor 15 (GDF-15) | Q99988 | Peptidoglycan recognition protein 1 (PGLYRP1) | O75594 |

| Perlecan (PLC) | P98160 | Tissue factor pathway inhibitor (TFPI) | P10646 |
| --- | --- | --- | --- |
| Plasminogen activator inhibitor 1 (PAI) | P05121 | Tissue-type plasminogen activator (t-PA) | P00750 |
| Platelet endothelial cell adhesion molecule (PECAM-1) | P16284 | Transferrin receptor protein 1 (TR) | P02786 |
| Platelet-derived growth factor subunit A (PDGF subunit A) | P04085 | Trefoil factor 3 (TFF3) | Q07654 |
| Platelet glycoprotein VI (GP6) | Q9HCN6 | Trem-like transcript 2 protein (TLT-2) | Q5T2D2 |
| Proprotein convertase subtilisin/kexin type 9 (PCSK9) | Q8NBP7 | Tumor necrosis factor ligand superfamily member 13B (TNFSF13B) | Q9Y275 |
| Protein delta homolog 1 (DLK-1) | P80370 | Tumor necrosis factor receptor 1 (TNF-R1) | P19438 |
| P-selectin (SELP) | P16109 | Tumor necrosis factor receptor 2 (TNF-R2) | P20333 |
| Pulmonary surfactant-associated protein D (PSP-D) | P35247 | Tumor necrosis factor receptor superfamily member 6 (FAS) | P25445 |
| Resistin (RETN) | Q9HD89 | Tumor necrosis factor receptor superfamily member 10C (TNFRSF10C) | O14798 |
| Retinoic acid receptor responder protein 2 (RARRES2) | Q99969 | Tumor necrosis factor receptor superfamily member 14 (TNFRSF14) | Q92956 |
| Scavenger receptor cysteine-rich type 1 protein M130 (CD163) | Q86VB7 | Tyrosine-protein kinase receptor UFO (AXL) | P30530 |
| Secretoglobin family 3A member 2 (SCGB3A2) | Q96PL1 | Tyrosine-protein phosphatase non-receptor type substrate 1 (SHPS-1) | P78324 |
| Spondin-1 (SPON1) | Q9HCB6 | Urokinase plasminogen activator surface receptor (U-PAR) | Q03405 |
| ST2 protein (ST2) | Q01638 | Urokinase-type plasminogen activator (uPA) | P00749 |
| Tartrate-resistant acid phosphatase type 5 (TR-AP) | P13686 | von Willebrand factor (vWF) | P04275 |
